# LAIPT: Lysine Acetylation Site Identification with Polynomial Tree

**DOI:** 10.3390/ijms20010113

**Published:** 2018-12-29

**Authors:** Wenzheng Bao, Bin Yang, Zhengwei Li, Yong Zhou

**Affiliations:** 1 School of Information and Electrical Engineering, Xuzhou University of Technology, Xuzhou 221018, China; 2 School of Information Science and Engineering, Zaozhuang University, Zaozhuang 277100, China; 3 School of Computer Science and Technology, China University of Mining and Technology, Xuzhou 221116, China; Zhengwei@126.com (Z.L.); yongzhou@163.com (Y.Z.)

**Keywords:** post-translational modification, flexible neural tree, polynomial feature forms

## Abstract

Post-translational modification plays a key role in the field of biology. Experimental identification methods are time-consuming and expensive. Therefore, computational methods to deal with such issues overcome these shortcomings and limitations. In this article, we propose a lysine acetylation site identification with polynomial tree method (LAIPT), making use of the polynomial style to demonstrate amino-acid residue relationships in peptide segments. This polynomial style was enriched by the physical and chemical properties of amino-acid residues. Then, these reconstructed features were input into the employed classification model, named the flexible neural tree. Finally, some effect evaluation measurements were employed to test the model’s performance.

## 1. Introduction

Post-translational modification (PTM) is one of the most significant processes in the field of biology. More than 650 types of post-translational modification were reported across several decades of efforts. Among these types of post-translational modification, several modifications have the ability to reverse their processes. PTM provides a fine-tuned control of protein function in various types of cells in the field of disease research and drug design [1,2,3,4]. For example, the well-known tumor suppressor p53 is subject to many post-translational modifications, which have the ability to alter its localization, stability, and other related functions, thus ultimately modulating its response to various forms of genotoxic stress [5,6,7,8,9,10]. Therefore, p53 drives both the activation and repression of a large number of promoters, which ultimately define its tumor suppressor abilities. This tumor suppressor is a critical transcription factor in the field of post-translational modification [11]. With these reversible modifications, protein structures change and their functions are enriched to some degree. As one of the most typical and classical reversible types of modification, lysine acetylation was reported about half a century ago [1,2]. Acetylation occurs on the ε-amino group of lysine residues; it was noted that three enzymes take part in this process. Whereas lysine deacetylases (KDACs) remove the acetyl groups of proteins, lysine acetyl transferases (KATs) transfer the acetyl group across proteins [3,4,5,6]. Considering the key role of lysine acetylation in several diseases and novel drug designations, a great deal of experimental approaches were proposed and introduced to identify the acetylation sites of lysine residues in protein sequences. These experimental approaches, including radioactivity chemical methods, chromatin immune precipitation (ChIP), and mass spectrometry, play their roles in various degrees [7,8]. Unfortunately, these experimental methods can hardly meet the need of identifying sites, and they are time-consuming and expensive. Considering this issue, effective identification methods, based on a computational biology approach, are urgently needed to identify acetylation modification sites, especially with the increasingly number of protein resources. 

When it comes to computational biology methods, several classical methods were introduced in the field of protein sequence procession [12,13,14,15,16]. Meanwhile, with the development of machine learning and artificial intelligence, some computational methods were proposed and designed to deal with similar issues at the DNA, RNA and protein levels [17,18,19]. Several milestone efforts were demonstrated in the field of identification of lysine modification sites. For instance, Xu et al. made use of a support vector machine (SVM) to identification lysine acetylation sites with ensemble information [20]. PLMLA(prediction of lysine methylation and lysine acetylation by combining multiple features), which was designed by Shi et al. in 2012, utilized information about protein sequences and secondary structure to demonstrate whether lysine residues were modified or not [21]. In the same year, PSKAcePred (Position-Specific Analysis and Prediction for Protein Lysine Acetylation Based on Multiple Features), which was proposed by Suo et al., was based on amino-acid composition and physicochemical properties to quantify protein segments [22]. Meanwhile, Shao et al. proposed BRABSB (bi-relative adapted binomial score Bayes), which made use of binomial score Bayesian [23]. Since then, SSPKA (species-specific lysine acetylation prediction), based on the random forest (RF) model, was proposed in 2014 to deal with such modification sites. Two years later, Wu et al. designed a novel approach named KA-predictor (Improved Species-Specific Lysine Acetylation Site Prediction) that utilized many different kinds of features to identify cases of lysine modification [24]. Overall, models for the effective identification of modification sites consist of two parts. The first part is feature description, which focuses on an effective method of showing protein sequence information or peptide segment information in several different aspects. The second part is the construction of the machine learning model, which aims to deal with different types of protein sequences or peptide segments with high accuracy and generalizability. The abovementioned methods, among others (PLMLA, Phosida, LysAcet, EnsemblePail, PSKAcePred, BRABSB, and SSPKA), can be regarded as the state of the art in this field.

Relationships among amino-acid residues need to be effectively described at the protein level. These relationships have the ability to demonstrate the local information of amino-acid residues in some peptide segments, and can be helpful in constructing more useful information with regards to the identification of modification sites. Some related work was proposed in DNA and RNA analysis [25,26,27,28,29,30,31]; methods such as DeepBind and DeepSea take advantage of deep convolutional neural networks (CNNs) to predict the sequence specificities of DNA-binding proteins [32,33,34,35]. In summary, these sequence analysis methods can be regarded as issues resolved using computational biology. 

When it comes to the abovementioned issues, Chou proposed five steps for dealing with them [35,36,37,38]. In the first step, available benchmark datasets should be selected, which are used to train and test machine learning models. In the second step, available methods for sequence quality expression should be selected. In the third step, an available algorithm should be used to identify positive and negative samples. In the fourth step, validation methods for evaluating the performances of the proposed methods should be selected. In the final step, a web resource should be constructed to detail the workflow, along with related raw data. Therefore, in this paper, we introduce a method for the identification of lysine acetylation sites following these steps.

In this article, we propose lysine acetylation site identification with polynomial tree method (LAIPT), making use of the polynomial style to demonstrate amino-acid residue relationships in peptide segments. This polynomial style was enriched by the physico-chemical properties of amino-acid residues. Then, these reconstructed features were input into the employed classification model, named the flexible neural tree (FNT). Finally, some effect evaluation measurements were employed to test the model’s performance. And the website of this work is shown in http://121.250.173.184/.

## 2. Results and Discussions

### 2.1. Comparison with Other Features

In order to evaluate the performance of the polynomial form features, several state-of-the-art methods were chosen for comparison, including binary encoding, amino acid composition (AA composition), grouping AA composition, physico-chemical property, k nearest neighbor features, and secondary tendency structure. The details of these comparisons are shown in Table 1, Table 2 and Table 3.

### 2.2. Comparison with Other Models

In order to more objectively evaluate the performance of the proposed feature description and employed classification model, we compared it with several state-of-the-art methods, including DBD (Breakdowns of B)-Threader, iDNA-Prot, and other similar tools in the field of sequence classification and post-translational modification. Details of these comparisons are shown in Table 4, Table 5 and Table 6.

**Figure 1:**
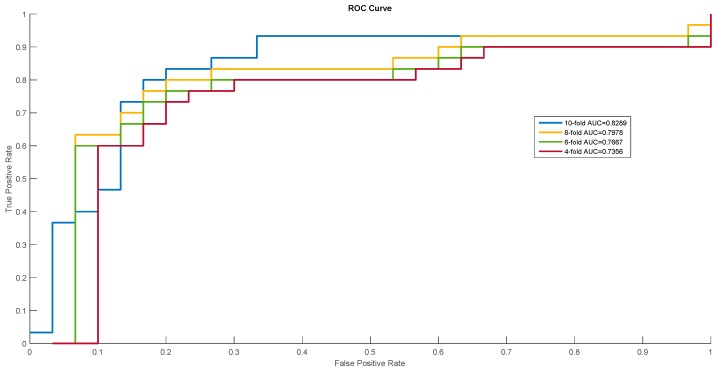
Receiver operating characteristic (ROC) curves of *Homo sapiens*.

**Figure 2:**
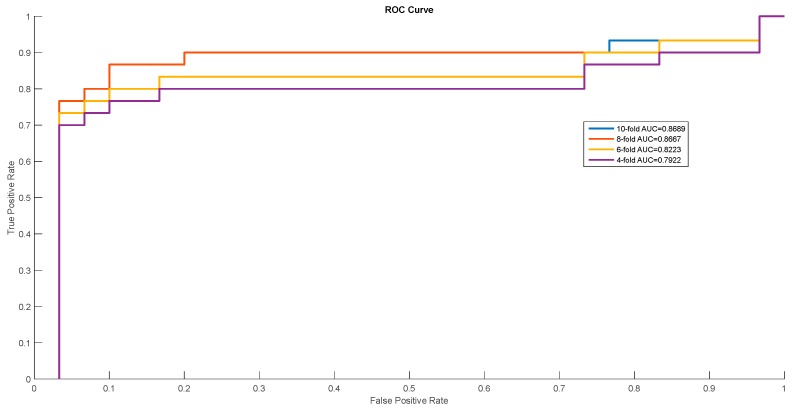
ROC curves of *Mus musculus*.

**Figure 3:**
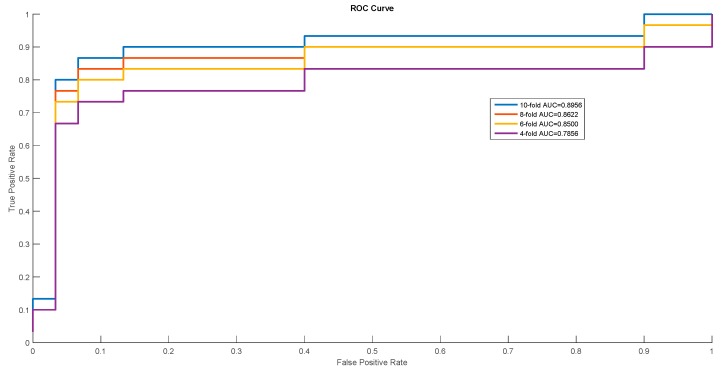
ROC Curves of *Escherichia coli*.

In order to show the proposed model’s stability and generalization, we utilized the ROC (receiver operating characteristic curve) curve to show the classification results. Meanwhile, some cross-validation methods (fourfold, sixfold, eightfold, and 10-fold) were also utilized. The detailed ROC curves for each species are shown in Figure 1, Figure 2 and Figure 3.

### 2.3. Performance Using Differences Bandwidths

In this work, the bandwidths of sliding windows played a significant role in the feature size. On the one hand, the lack of a bandwidth can waste computational resources and result in ineffective feature description. On the other hand, different species may have unique bandwidths in this classification model. Therefore, we tested bandwidths ranging from 21 to 31, with an interval of 2. Detailed results for each of these bandwidths in the selected species are shown in Table 7. In order to more objectively show the results, we compared them with other machine learning methods, including SVM, NN, and RF.

From the above table, we can easily determine that the most appropriate bandwidths for *Homo sapiens*, *Mus musculus*, and *Escherichia coli* were 25, 25, and 23, respectively Furthermore, the FNT model performed better than the three other machine learning methods in the majority of measurements among these bandwidths.

### 2.4. Performance of Polynomial Feature Description

In this section, we discuss the parameter selection of polynomial feature description. The three proposed feature description methods constitute five parameters, one of which is described by Equation (1), two of which are described by Equation (2), and two of which are described by Equation (3). The three proposed methods were compared, taking into account the coefficients *a*_1_ and *a*_2_, and the constants *b*_1_, *b*_2_, and *c*. We defined *a*_1_ and *a*_2_ in the range [−10, 10], and three constants in the range [−100, 100], to test the performance of the employed classification method. We determined that the most appropriate parameters of the three proposed features were as follows: *c* = 57.6, *a*_1_ = 4.1, *a*_2_ = −2.7, *b*_1_ = 27.1, and *b*_2_ = 67.1. The three proposed features performed differently. The abovementioned classification models were also used for comparison. In order to reduce the usage of unnecessary computational resources, the most appropriate bandwidths determined previously were used. The Details of the results are shown in Table 8.

(1) $$y=c_{1}$$

(2) $$y=a_{1}x^{\frac{1}{2}}+b_{1}$$

(3) $$y=a_{2}x^{2}+b_{2}$$

From the above table, we can easily determine that the different features performed differently. The most appropriate feature description was that described by Equation (3) for both *H. sapiens* and *E. coli*, while that described by the Equation (2) was most suitable for *M. musculus*. 

## 3. Materials and Methods

**Figure 4:**
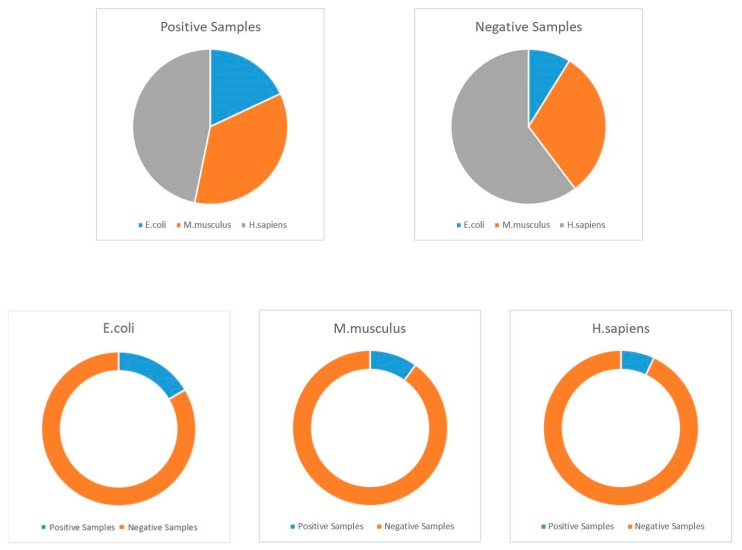
Distribution of employed datasets.

Because of the ubiquity and universality of lysine acetylation at the protein level, we can find several acetylated proteins in various databases, including NCBI (National Center for Biotechnology Information), Uniprot, and other related proteomics databases. In this study, we selected about 30,000 protein sequences, which contain more than 111,200 acetylation sites among them [49]. These proteins could be extracted from the Protein Lysine Modification Database (PLMD) version 3.0 [50]. PLMD is one of the most well-known and commonly used post-translational modification site databases, and it contains more than 20 types of lysine modification in more than 170 species at the protein level. Generally, this database can be treated as the largest available acetylation database; thus, it was employed as the benchmark dataset in this work. Unfortunately, overestimation may be one of the most significant limitations when using machine learning. In order to overcome this shortcoming, CD-HIT (Cluster Database at High Identity with Tolerance) was utilized to remove some homologous sequences [51,52,53,54]. In this work, we utilized a threshold of 40% similarity with this tool. Following this process, we obtained 59,532 proven acetylated modification sites from 20,527 protein sequences. These protein data were used to construct the training, testing, and independent datasets. During this classification process, we defined the proven acetylated sites as positive samples and the non-proven modifications as negative samples. Detailed information of the employed datasets is shown in Table 9, and details with regards to the construction of datasets are shown in Figure 4.

In this work, we employed the general dataset as the training and testing datasets. In order to evaluate the generalization and stability, we employed three species incorporating lysine acetylation sites as the independent datasets.

After constructing the available datasets, some peptide segments were extracted from the whole protein sequences. In order to reduce the unnecessary usage of storage space and computational resources, some peptides with a central lysine residue were extracted in this work. We made use of sliding windows to extract peptide segments with a size of 2*n* + 1 [55], where *n* is the length of the upstream or downstream fragment, and 1 is the position of the central lysine residue in the segment. In this work, the length of the upstream fragment was equal to that of the downstream fragment, and *n* ranged from 10 to 15. Thus, the whole length of the sliding window was between 21 and 31. In the next section, we discuss the performances of the various selected lengths of sliding window.

### 3.1. Encoding of Protein Fragments

Several different types of features for quantifying biological sequences were presented across many years of protein research, such as amino-acid composition, position special scoring matrix, physico-chemical properties, and other related features [56,57,58] These features can demonstrate sequence information in various aspects, and they play various roles in protein sequence analysis. However, few features can demonstrate the relationships of amino-acid residues. In this paper, each peptide was treated as a sample. According to biological concepts, neighboring amino-acid residues present both coordination and individual functions. On this basis, we tried utilizing some of these functions to describe the relationships in this work.

**Figure 5:**
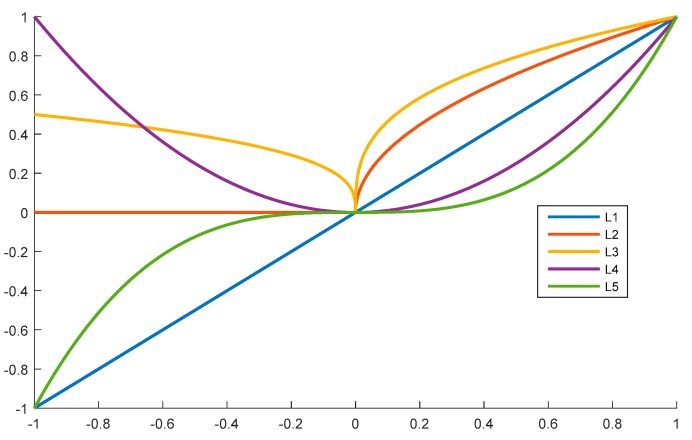
Curves of Equations (4)–(8) in the range [−1, 1].

We propose a polynomial method to describe the relationships between the central lysine residue and the neighboring amino-acid residues. Several forms of polynomial styles exist, such as the constant form, linear function form, quadratic function form, cubic function form, and so on. For example, we show the curves of these four forms in Figure 5.

In Figure 5, L1, L2, L3, L4, and L5 follow Equations (4)–(8), respectively.

(4) y=x

(5) $$y=x^{\frac{1}{2}}$$

(6) $$y=x^{\frac{1}{3}}$$

(7) $$y=x^{2}$$

(8) $$y=x^{3}$$

From Figure 5, we can easily determine that both L2 and L4 are even functions, while the other curves are odd functions. Considering that the upstream and the downstream fragments played the same role in the selected peptide segments, the even functions were selected for this work; therefore, we utilized three types of functions. The first one was the constant function, whereby all amino-acid residues in the peptide segments have the same influence, as described in Equation (1). The second function followed Equation (2), and the third function followed the Equation (3).

$$y=c_{1}$$

$$y=a_{1}x^{\frac{1}{2}}+b_{1}$$

$$y=a_{2}x^{2}+b_{2}$$

where the parameters *a*_1_, *a*_2_, *b*_1_, *b*_2_, and *c*_1_ were optimized in this work. It was noted that both Equations (1) and (2) could hardly be described as linear functions. Thus, the center of the last two functions was designated as the origin point, i.e., the classified modification sites in the peptide segments. Regions to the left and right part of this origin point were designated as the upstream and the downstream segments, respectively. The influence of each neighboring amino-acid residue is defined below.

According to Equation (1), the relationship between a neighboring amino-acid residue and the central lysine is shown in Equation (9).

(9) $$influ_{1}=[c_{1},c_{1},\cdots ,c_{1}]$$

where influ1 contains 2*n* + 1 elements in each sample, and *c*_1_ is the relationship between each amino-acid residue in the selected peptide segment. In this function, every amino-acid residue has the same influence; thus, the amino-acid composition can be regarded as a special form of this style.

According to Equation (2), the relationship between the neighboring and central residues are shown in Equation (10).

(10) $$influ_{2}=[a_{1}n^{\frac{1}{2}}+b_{1},\cdots a_{1}+b_{1},b_{1},a_{1}+b_{1}\cdots ,a_{1}n^{\frac{1}{2}}+b_{1}]$$

where influ2 also contains 2*n* + 1 elements, and each value of influ2 follows the discrete values of Equation (2) and has the range [−*n*, *n*].

According to the Equation (3), the relationship between two amino-acid residues is shown in Equation (11).

(11) $$influ_{3}=[a_{1}n^{2}+b_{1},\cdots a_{1}+b_{1},b_{1},a_{1}+b_{1}\cdots ,a_{1}n^{2}+b_{1}]$$

where he influ3 also contains 2*n* + 1 elements, and each value of influ3 follows the discrete values of Equation (11) and has the range [−*n*, *n*].

After demonstrating the fundamental relationship of amino-acid residues within the classified peptide, the next step was to enrich the related properties of amino-acid residues. In this step, physical, chemical, evolutional, structural, and other related information was enriched using the three styles proposed above.

### 3.2. Physico-Chemical Properties

Physico-chemical properties are widely and successfully utilized in the identification of protein post-translational modifications, including ubiquitination, phosphorylation, and others [59,60]. These properties can help determine the fundamental characteristics of proteins in several aspects. One of the most well-known and widely utilized databases is AAIndex [61,62], which contains a great deal of physico-chemical and biochemical information for each amino-acid residue and some amino-acid compositions. The latest version of this database describes 544 properties of amino acid residues. Among these properties, following previous efforts and research [62], we selected several of them, which are listed in Table 10.

Considering the abovementioned elements, we minimized the presence of useless information; therefore, the area under the receiver operating characteristic (ROC) curve (AUC) was used to evaluate the measurements in this work.

### 3.3. Prediction Algorithm

**Figure 6:**
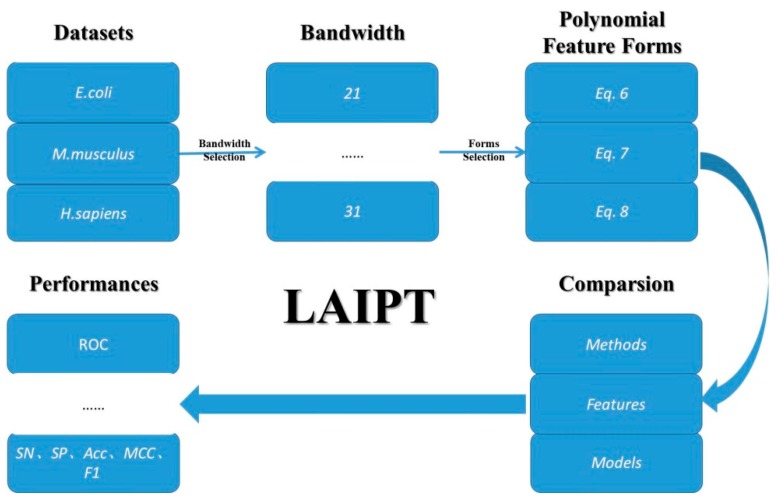
The outline of the lysine acetylation site identification with polynomial tree method (LAIPT).

The computational identification of modification sites focuses on classification models in the field of machine learning. In this thesis, we employed machine learning models, including the flexible neural tree. We employed three machine learning methods for the three elements in the classification. The first element involved the bandwidth of the sliding windows in the classified peptide segments, the second involved the parameters of polynomial feature description, and the third involved the selection of different combinations. Therefore, the classification model was designed to deal with these three elements; the detailed outline of this algorithm is demonstrated in Figure 6.

**Figure 7:**
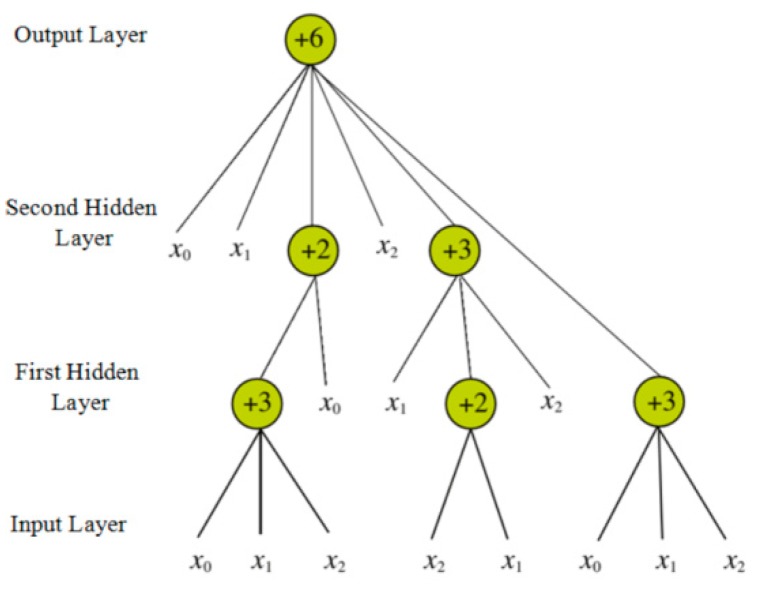
Typical structure of a flexible neural tree (FNT).

The flexible neural tree (FNT) was proposed by Chen [63,64], and it can be treated as an alternative tree neural network. Therefore, this model can be utilized to deal with the issues of classification and prediction in the field of machine learning. The typical structure of an FNT is shown in Figure 7. 

From the above figure, we can easily determine that the model contains three types of layers—the input layer, the hidden layer, and the output layer. The network function of this model is shown in Equations (12) and (13).

(12) $$network_{i}=\sum_{j=1}^{i}\omega_{j}\times y_{j}$$

(13) $$out_{i}=f(m_{i},n_{i},network_{i})=e^{-{\left(\frac{network_{i}-m_{i}}{n_{i}}\right)}^{2}}$$

where *w_j_* is the weight of the *j*-th input element, and *y_j_* is the *j*-th element of the input sample. Both *m_i_* and *n_i_* are parameters in this network. 

### 3.4. Performance Measurements

Some well-known methods exist in the field of machine learning for evaluating performance measurements. In this work, some typical measurements, including sensitivity, specificity, accuracy, F1 scores, and Matthew’s correlation coefficients (MCCs) [65,66], of the identified modification sites were used. Furthermore, the AUC [67] was also employed to test the performance of imbalanced classification problems, whereby the negative sample size was much bigger than the positive sample size. 

In this classification problem, samples can be defined as two types—positive samples and negative samples. Positive samples refer to peptide segments where the central lysine is acetylated, while negative samples refer to peptide segments where the central lysine is not. According to the definitions of the classified samples, there can be four outcomes. If a positive sample is classified as true, this can be deemed a true positive (*TP*). If a positive sample is classified as false, this can be deemed a false positive (*FP*). Following this concept, a negative sample classified as true is a true negative (*TN*), and a negative sample classified as false is a false negative (*FN*). According to the number of *TP*, *TN*, *FP*, and *FN*, we can easily obtain measures of sensitivity, specificity, accuracy, F1 scores, and MCC.

(14) $$Accuracy=\frac{TP+TN}{P+N}$$

(15) $$Sensitivity=\frac{TP}{TP+FN}$$

(16) $$Specificity=\frac{TN}{TN+FP}$$

(17) $$F1=\frac{2TP}{2TP+FN+FP}$$

(18) $$MCC=\frac{TP\times TN-FP\times FN}{\sqrt{\left(TP+FP)(TP+FN)(TN+FP)(TN+FN\right)}}$$

where *P* is the number of positive samples and *N* is the number of negative samples. Nevertheless, in Equations (14)–(18), there is a lack of intuitiveness, and they can hardly be described as easy to understand for the majority of researchers in the field of biology. The interpretation of MCC in particular is not at all intuitive in this form, although this measurement plays a key role in the evaluation of the classification model’s stability. Therefore, we made use of the concept based on Chou, proposed at the beginning of this century. In this concept, the total number of positive samples can be defined as *N*^+^, and the total number of negative samples can be defined as the *N*^−^. Then, the number of misclassified positive samples can be treated as the $N_{-}^{+}$, and the number of misclassified negative samples can be treated as the $N_{+}^{-}$. With this definition, *TP*, *TN*, *FP*, and *FN* can be described in Equations (19)–(22).

(19) $$TP=N_{}^{+}-N_{-}^{+}$$

(20) $$TN=N_{}^{-}-N_{+}^{-}$$

(21) $$FP=N_{+}^{-}$$

(22) $$FN=N_{-}^{+}$$

Thus, the abovementioned measurements can be newly defined as Equations (23)–(27).

(23) $$Accuracy=1-\frac{N_{-}^{+}+N_{+}^{-}}{N^{+}+N^{-}}$$

(24) $$Sensitivity=1-\frac{N_{-}^{+}}{N^{+}}$$

(25) $$Specificity=1-\frac{N_{+}^{-}}{N^{-}}$$

(26) $$F1=\frac{2(N^{+}-N_{-}^{+})}{2N^{+}-N_{-}^{+}+N_{+}^{-}}$$

(27) $$MCC=\frac{1-(\frac{N_{-}^{+}}{N^{+}}+\frac{N_{+}^{-}}{N^{-}})}{\sqrt{\left(1+\frac{N_{+}^{-}-N_{-}^{+}}{N^{+}})(1+\frac{N_{-}^{+}-N_{+}^{-}}{N^{-}}\right)}}$$

The interpretations of each performance metric in Equations (23)–(27) are far more intuitive and easier to understand for biological researchers. For instance, when samples can be correctly classified, whereby all positive samples are classified as true and all negative samples are classified as false, we get $N_{+}^{-}$ = 0 and $N_{-}^{+}$ = 0, and the sensitivity and specificity are both equal to 1. Meanwhile, the accuracy is equal to 1 and MCC is also equal to 1 in such a situation. On the contrary, if all positive samples are classified as false and all negative samples are classified as true, $N_{+}^{-}$ and $N_{-}^{+}$ are both equal to 1, and the sensitivity and specificity are both equal to 0. Furthermore, the accuracy is equal to 0, and the MCC is equal to −1 in this situation. In a random classification issue, $N_{+}^{-}$= 0.5*N*^−^ and $N_{-}^{+}$ = 0.5*N*^+^. Thus, the accuracy is equal to 0.5 and MCC is equal to 0 in this situation. This definition method has several advantages [68,69,70,71]; however, utilizing these five measurements can hardly meet required performance in a scenario of imbalanced classification. Therefore, we made use of ROC and precision recall. ROC can be shown by the relationship between the true positive rate (TPR) and the false positive rate (FPR) in the classification. Meanwhile, precision recall can be demonstrated by the relationship between the precision and recall. 

## 4. Conclusions

In this article, we proposed a lysine acetylation site identification with polynomial tree method (LAIPT), making use of the polynomial style to demonstrate the amino-acid residue relationships in peptide segments. The polynomial style was enriched by the physico-chemical properties of amino-acid residues. Then, these reconstructed features were input into the employed classification model, named the flexible neural tree. Finally, some effect evaluation measurements were employed to test the model’s performances. We demonstrated that the three employed species modification sites constituted unique feature descriptions. In the future, we hope to determine more useful forms of feature description and to utilize effective classification models to deal with them. We hope that the algorithm described herein has the ability to deal with other types of protein post-translational modification sites in various species.

## Figures and Tables

**Table 1:** Performances of different features in *E. coli*. Sn—sensitivity; Sp—specificity; Acc—accuracy; MCC—Matthew’s correlation coefficient.

Features	Sn (%)	Sp (%)	Acc (%)	F1	MCC
Binary encoding	43.36	75.80	59.58	0.5175	0.2026
AA composition	64.14	52.79	58.46	0.6070	0.1704
Grouping AA composition	41.78	76.04	58.91	0.5042	0.1897
Physico-chemical properties	55.53	63.93	59.73	0.5796	0.1953
KNN features	64.94	55.85	60.39	0.6212	0.2088
Secondary tendency structure	59.96	57.40	58.68	0.5920	0.1737
PSSM	51.20	69.39	60.30	0.5632	0.2094
Proposed algorithm	73.26	74.01	73.63	0.7354	0.4727

**Table 2:** Performances of different features in *M. musculus*.

Features	Sn (%)	Sp (%)	Acc (%)	F1	MCC
Binary encoding	42.27	75.38	58.82	0.5065	0.1871
AA composition	63.05	52.37	57.71	0.5985	0.1551
Grouping AA composition	40.69	75.62	58.15	0.4930	0.1741
Physico-chemical properties	54.44	63.51	58.97	0.5702	0.1802
KNN features	63.85	55.43	59.64	0.6127	0.1935
Secondary tendency structure	58.87	56.98	57.92	0.5832	0.1585
PSSM	50.11	68.97	59.54	0.5533	0.1943
Proposed algorithm	73.52	74.31	73.91	0.7381	0.4783

**Table 3:** Performances of different features in *H. sapiens*.

Features	Sn (%)	Sp (%)	Acc (%)	F1	MCC
Binary encoding	43.14	74.74	58.94	0.5123	0.1885
AA composition	63.92	51.73	57.83	0.6025	0.1577
Grouping AA composition	41.56	74.98	58.27	0.4990	0.1755
Physico-chemical properties	55.31	62.87	59.09	0.5748	0.1823
KNN features	64.72	54.79	59.76	0.6166	0.1961
Secondary tendency structure	59.74	56.34	58.04	0.5874	0.1609
PSSM	50.98	68.33	59.66	0.5582	0.1961
Proposed algorithm	74.82	72.42	73.62	0.7393	0.4725

**Table 4:** Performances of different methods in *E. coli*.

Method	Sn (%)	Sp (%)	Acc (%)	F1	MCC
DNABIND [39]	65.78	67.97	66.88	0.6651	0.3376
DNAbinder [39]	56.87	63.79	60.33	0.5891	0.2071
DBD-Threader [40]	22.79	94.71	58.75	0.3559	0.2519
DNA-Prot [40]	67.81	53.71	60.76	0.6334	0.2174
iDNA-Prot [40]	65.71	65.72	65.72	0.6571	0.3143
DBPPred [41]	75.37	72.87	74.12	0.0811	0.4826
PLMLA [42]	60.80	64.70	62.70	0.4748	0.2550
Phosida [43]	70.61	54.90	62.70	0.6547	0.2580
LysAcet [44]	27.50	76.50	52.00	0.3642	0.0450
EnsemblePail [45]	27.50	66.70	47.10	0.3420	-0.0640
PSKAcePred [46]	41.20	60.80	51.00	0.4568	0.0200
BRABSB [47]	51.00	60.80	55.90	0.5363	0.1180
SSPKA [48]	54.90	76.50	65.70	0.6155	0.3210
Proposed algorithm	73.26	74.01	73.63	0.7354	0.4727

**Table 5:** Performances of different methods in *M. musculus*.

Method	Sn (%)	Sp (%)	Acc (%)	F1	MCC
DNABIND [39]	63.31	64.54	63.93	0.6370	0.2785
DNAbinder [39]	58.14	65.54	61.84	0.6037	0.2375
DBD-Threader [40]	26.54	92.45	59.50	0.3959	0.2525
DNA-Prot [40]	69.21	58.43	63.82	0.6567	0.2780
iDNA-Prot [40]	69.54	66.45	68.00	0.6848	0.3601
DBPPred [41]	78.45	74.45	76.45	0.7691	0.5294
PLMLA [42]	51.60	51.90	51.70	0.5168	0.0350
Phosida [43]	59.00	54.60	56.80	0.5773	0.1370
LysAcet [44]	43.10	67.00	55.00	0.4895	0.1040
EnsemblePail [45]	51.10	76.20	63.50	0.5843	0.2820
PSKAcePred [46]	51.10	65.90	58.40	0.5518	0.1720
BRABSB [47]	63.80	58.40	61.10	0.6212	0.2220
SSPKA [48]	64.80	66.50	65.70	0.6536	0.3140
Proposed algorithm	73.52	74.31	73.91	0.7381	0.4783

**Table 6:** Performances of different methods in *H. sapiens*.

Method	Sn (%)	Sp (%)	Acc (%)	F1	MCC
DNABIND [39]	65.72	67.42	66.57	0.6628	0.3314
DNAbinder [39]	57.87	66.97	62.42	0.6063	0.2494
DBD-Threader [40]	27.28	90.63	58.96	0.3993	0.2315
DNA-Prot [40]	66.74	60.74	63.74	0.6480	0.2753
iDNA-Prot [40]	67.54	65.79	66.67	0.6695	0.3334
DBPPred [41]	79.74	73.85	76.80	0.7746	0.5368
PLMLA [42]	63.00	66.30	64.80	0.6406	0.2960
Phosida [43]	55.30	58.30	56.80	0.5614	0.1360
LysAcet [44]	50.30	61.60	55.80	0.5331	0.1200
EnsemblePail [45]	45.70	61.80	53.50	0.4970	0.0760
PSKAcePred [46]	55.30	55.80	55.60	0.5544	0.1110
BRABSB [47]	61.20	66.30	63.70	0.6280	0.2750
SSPKA [48]	48.20	72.50	60.00	0.5487	0.2140
Proposed algorithm	74.82	72.42	73.62	0.7393	0.4725

**Table 7:** Performance using different bandwidths. SVM—support vector machine; NN—neural network; RF—random forest.

Species	Bandwidth	Method	Sn (%)	Sp (%)	Acc (%)	F1	MCC
*H. sapiens*	21	SVM	70.34	66.52	68.43	0.6902	0.3689
		NN	67.67	65.72	66.70	0.6702	0.3340
		RF	72.37	68.61	70.49	0.7103	0.4101
		FNT	70.51	70.87	70.69	0.7064	0.4138
	23	SVM	66.48	65.89	66.18	0.6628	0.3237
		NN	63.81	65.09	64.45	0.6422	0.2890
		RF	68.51	67.98	68.24	0.6833	0.3649
		FNT	66.65	70.24	68.44	0.6787	0.3691
	25	SVM	72.28	68.43	70.36	0.7092	0.4075
		NN	69.61	67.63	68.62	0.6893	0.3725
		RF	74.31	70.52	72.42	0.7293	0.4487
		FNT	72.45	72.78	72.62	0.7257	0.4524
	27	SVM	69.38	65.94	67.66	0.6821	0.3534
		NN	66.71	65.14	65.92	0.6619	0.3185
		RF	71.41	68.03	69.72	0.7022	0.3946
		FNT	69.55	70.29	69.92	0.6981	0.3984
	29	SVM	69.71	66.31	68.01	0.6854	0.3604
		NN	67.04	65.51	66.27	0.6653	0.3255
		RF	71.74	68.40	70.07	0.7056	0.4016
		FNT	69.88	70.66	70.27	0.7015	0.4054
	31	SVM	68.36	64.23	66.30	0.6698	0.3262
		NN	65.69	63.43	64.56	0.6496	0.2913
		RF	70.39	66.32	68.36	0.6899	0.3674
		FNT	68.53	68.58	68.56	0.6855	0.3711
*M. musculus*	21	SVM	69.99	69.11	69.55	0.6968	0.3910
		NN	67.32	65.31	66.32	0.6665	0.3264
		RF	71.02	70.20	70.61	0.7073	0.4122
		FNT	72.37	73.36	72.87	0.7273	0.4573
	23	SVM	69.09	67.70	68.40	0.6861	0.3679
		NN	66.42	63.90	65.16	0.6560	0.3033
		RF	70.12	68.79	69.46	0.6966	0.3891
		FNT	71.47	71.95	71.71	0.7164	0.4342
	25	SVM	71.06	69.95	70.51	0.7067	0.4102
		NN	68.39	66.15	67.27	0.6763	0.3455
		RF	72.09	71.04	71.57	0.7171	0.4313
		FNT	73.44	74.20	73.82	0.7372	0.4764
	27	SVM	70.98	69.67	70.32	0.7052	0.4065
		NN	68.31	65.87	67.09	0.6749	0.3419
		RF	72.01	70.76	71.38	0.7156	0.4277
		FNT	73.36	73.92	73.64	0.7356	0.4728
	29	SVM	70.37	69.52	69.95	0.7007	0.3989
		NN	67.70	65.72	66.71	0.6704	0.3343
		RF	71.40	70.61	71.01	0.7112	0.4201
		FNT	72.75	73.77	73.26	0.7312	0.4652
	31	SVM	70.23	69.47	69.85	0.6997	0.3970
		NN	67.56	65.67	66.62	0.6693	0.3324
		RF	71.26	70.56	70.91	0.7101	0.4182
		FNT	72.61	73.72	73.17	0.7302	0.4634
*E. coli*	21	SVM	68.53	68.92	68.73	0.6867	0.3745
		NN	65.86	65.12	65.49	0.6562	0.3098
		RF	68.56	70.01	69.29	0.6906	0.3858
		FNT	70.70	72.27	71.49	0.7126	0.4298
	23	SVM	70.82	69.56	70.19	0.7038	0.4038
		NN	68.15	65.76	66.96	0.6735	0.3392
		RF	70.85	70.65	70.75	0.7078	0.4150
		FNT	72.99	72.91	72.95	0.7296	0.4590
	25	SVM	71.03	69.86	70.45	0.7062	0.4090
		NN	68.36	66.06	67.21	0.6758	0.3443
		RF	71.06	70.95	71.01	0.7102	0.4201
		FNT	73.20	73.21	73.21	0.7320	0.4641
	27	SVM	70.36	69.60	69.98	0.7009	0.3996
		NN	67.69	65.80	66.74	0.6706	0.3349
		RF	70.39	70.69	70.54	0.7049	0.4108
		FNT	72.53	72.95	72.74	0.7268	0.4548
	29	SVM	70.12	69.28	69.70	0.6982	0.3939
		NN	67.45	65.48	66.46	0.6679	0.3293
		RF	70.15	70.37	70.26	0.7022	0.4051
		FNT	72.29	72.63	72.46	0.7241	0.4491
	31	SVM	69.56	68.81	69.18	0.6930	0.3837
		NN	66.89	65.01	65.95	0.6627	0.3190
		RF	69.59	69.90	69.74	0.6970	0.3949
		FNT	71.73	72.16	71.94	0.7188	0.4389

**Table 8:** Performance of different functions.

Species	Function	Method	Sn (%)	Sp (%)	Acc (%)	F1	MCC
*H. sapiens*	Equation (1)	SVM	72.28	68.43	70.36	0.7091	0.4074
		NN	69.61	67.63	68.62	0.6893	0.3725
		RF	72.28	68.43	70.36	0.7091	0.4074
		FNT	74.31	70.52	72.415	0.7293	0.4486
	Equation (2)	SVM	73.11	68.90	71.00	0.7160	0.4205
		NN	70.44	68.10	69.27	0.6963	0.3855
		RF	73.11	68.90	71.00	0.7160	0.4205
		FNT	75.14	70.99	73.06	0.7361	0.4617
	Equation (3)	SVM	72.79	70.33	71.56	0.7191	0.4313
		NN	70.12	69.53	69.82	0.6991	0.3965
		RF	72.79	70.33	71.56	0.7191	0.4313
		FNT	74.82	72.42	73.62	0.7393	0.4725
*M. musculus*	Equation (1)	SVM	71.06	69.95	70.51	0.7067	0.4101
		NN	68.39	66.15	67.27	0.6763	0.3455
		RF	72.09	71.04	71.57	0.7171	0.4313
		FNT	73.44	74.20	73.82	0.7372	0.4764
	Equation (2)	SVM	71.14	70.06	70.60	0.7076	0.4120
		NN	68.47	66.26	67.36	0.6772	0.3474
		RF	72.17	71.15	71.66	0.7180	0.4332
		FNT	73.52	74.31	73.91	0.7381	0.4783
	Equation (3)	SVM	71.16	70.62	70.89	0.7097	0.4179
		NN	68.49	66.82	67.66	0.6793	0.3532
		RF	72.19	71.71	71.95	0.7202	0.4391
		FNT	73.54	74.87	74.21	0.7404	0.4842
*E. coli*	Equation (1)	SVM	70.82	69.56	70.19	0.7038	0.4038
		NN	68.15	65.76	66.96	0.6735	0.3392
		RF	70.85	70.65	70.75	0.7078	0.4150
		FNT	72.99	72.91	72.95	0.7296	0.4590
	Equation (2)	SVM	71.32	70.09	70.70	0.7088	0.4141
		NN	68.65	66.29	67.47	0.6785	0.3495
		RF	71.35	71.18	71.26	0.7129	0.4253
		FNT	73.49	73.44	73.46	0.7347	0.4693
	Equation (3)	SVM	71.09	70.66	70.87	0.7094	0.4175
		NN	68.42	66.86	67.64	0.6789	0.3528
		RF	71.12	71.75	71.43	0.7134	0.4287
		FNT	73.26	74.01	73.63	0.7354	0.4727

**Table 9:** Detailed information of employed datasets.

Dataset	Protein Sequences	Positive Samples	Negative Samples
General	15,575	55,773	863,365
*Homo sapiens*	1032	10,299	43,373
*Mus musculus*	343	3441	9788
*Escherichia coli*	143	2005	1528

**Table 10:** Selected properties from the AAIndex database. AA—amino acid.

Number	AAIndex ID	Name of Properties
1	CHOP780207	Normalized frequency of C-terminal non-helical region
2	DAYM780201	Relative mutability
3	EISD860102	Atom-based hydrophobic moment
4	FAUJ880108	Localized electrical effect
5	FAUJ880111	Positive charge
6	FINA910103	Helix termination parameter at position *j*-2, *j*-1, *j*
7	JANJ780101	Average accessible surface area
8	KARP850103	Flexibility parameter for two rigid neighbors
9	KLEP840101	Net charge
10	KRIW710101	Side-chain interaction parameter
11	KRIW790102	Fraction of site occupied by water
12	NAKH920103	AA composition of ejecta of single-spanning proteins
13	QIAN880101	Weights for alpha-helix at the window position of −6
